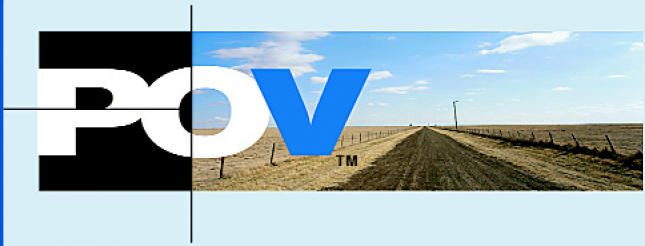# EHPnet: POV’s Borders: Environment

**Published:** 2004-12

**Authors:** Erin E. Dooley

The Public Broadcasting Service (PBS) has long been known for the quality of its programming, which runs the gamut from children’s shows to revealing documentaries. Now PBS is taking its talents to the Internet with an award-winning series, *POV’s Borders*. An outgrowth of PBS’s long-running television series *POV*, each yearly installment of the online series seeks to challenge visitors’ preconceptions about everyday aspects of our existence. The latest installment in the online series is *POV’s Borders: Environment*. Located at **http://www.pbs.org/pov/borders/2004/index.html**, the multimedia presentation uses a plethora of tools to explore how people relate to the three essential elements of our existence: air, water, and the soil that grows our food.

According to the Air section of the website, there are 31 million vehicles in California serving a population of 36 million people. This portion of the website looks at what drives Californians’ auto purchasing choices. There are video and print interviews with people who purchased electric and hybrid cars, an online chat room that allows visitors to voice their opinion about which type of vehicle is best for the environment, and a mini-documentary about the first service station in California to offer alternative fuels.

The Water section examines the debate in the United States over drinking bottled water versus tap water. Among the issues in this debate is the amount of plastic piling up as a result of bottled water consumption. In this section, one man tells how he reuses his water bottles. There is also a portion on America’s most polluted waterway, Newtown Creek, which runs between Brooklyn and Queens in New York City. A short film describes how children have worked to help clean up this desolate waterway and reclaim it as a natural space. The site includes tips to help visitors do their own waterway mapping and links to other sites that focus on water quality, such as the Environmental Protection Agency’s volunteer monitoring page.

The Earth section looks at the ground as a source of food. Two interactive features in this section teach visitors about heirloom varieties of plants and about saving seeds. There is also an interview with photographer and pasta maker Douglas Gayeton about the Slow Foods movement in Italy. This movement is trying to conserve traditional processes of raising animals and plants as well as producing food products.

Gayeton is also featured in Border Talk, one of three complementary sections of the site. The Border Talk section presents essays by artists, scientists, and others whose work is related to the environment. The For Educators section of the site provides six free lesson plans to accompany the Air, Water, and Earth pages. The PDF- and HTML-format lesson plans are suitable for middle school and high school classes. Finally, the Resources section provides a convenient index by category of all of the websites referenced throughout the site.

## Figures and Tables

**Figure:**